# Diallel analysis of cowpea (*Vigna unguiculata* (L.) Walp.) genotypes under water deficit stress

**DOI:** 10.1186/s12870-023-04508-0

**Published:** 2023-11-03

**Authors:** Vincent Ezin, Thibaut A. W. Tossou, Ifagbémi Bienvenue Chabi, Adam Ahanchede

**Affiliations:** 1 Department of Crop Production, Faculty of Agricultural Sciences, University of Abomey-Calavi, 01 BP 526 Cotonou, Benin; 2 Laboratory of Valorization and Quality Management of Food Bio-Ingredients, Faculty of Agricultural Sciences, University of Abomey-Calavi, 01 BP 526 Cotonou, Benin

**Keywords:** *Vigna unguiculata* L., Water deficit stress, Combinng ability, Diallel

## Abstract

Combining ability is referred to as the hybridization value of the parental genotypes involved in the crossing to develop hybrids. The best parents are selected through combining ability methods and subsequently used to produce high yielding and resistant hybrids. Thus, the objectives of this study were to (i) understand the nature and action of genes controlling water deficit tolerance, and (ii) identify superior genotypes from the genetic breadth provided by hybridization in cowpea. Twenty-four genotypes were subjected to normal irrigation and water deficit condition to examine combining ability, genotypic and phenotypic correlations for traits directly related to water deficit (proline and chlorophylls), grain yield and yield components. The results showed the presence of the action of additive and non-additive genes under both water regime conditions. However, there was the predominance of the action of additive genes for most of the traits studied under both conditions. The parents KVX61-1, IT06K242-3, IT07K-211–1-8, Kpodjiguèguè, IT99K-573–1-1, Tawa and IT97K-206–1-1 were observed to be good general combiners for proline content, chlorophyll content and traits associated with yield, while KVX61-1 × KVX396-18, IT06K242-3 × KVX396-18, IT07K-211–1-1 × KVX396-18, Kpodjiguèguè x KVX396-18, KVX61 -1 × IT97K-206–1-1, IT06K242-3 × IT97K-206–1-1, IT07K-211–1-1 × IT97K-206–1-1 and Kpodjiguèguè x IT97K-206–1-1 were proven to be the best specific combiners for traits directly related to water deficit tolerance and yield. It should be noted that number of days to pod maturity, pod length, number of pods per plant and weight of hundred seeds were highly heritable traits in this study.

## Introduction

In sub-Saharan Africa, cowpea (*Vigna unguiculata* (L.) Walp.) represents 64% of world production, i.e. 7.6 million tonnes per year and is therefore the most important indigenous legume [33]. Cowpea is mainly grown for human consumption as fresh, canned and dehydrated product because of its richness in proteins, carbohydrates, vitamins, minerals and dietary fiber [4, 13]. The highest consumption of cowpea is recorded in underdeveloped nations, where the population growth rates are utmost, further augmenting the demand for this indigenous valuable [23]. 96% of global cowpea production and consumption is in Sub-Saharan Africa [11]. Cowpea is a multipurpose, nitrogen-fixing and drought-tolerant crop [27]. Cowpea is one of the resilient crops that flourishes in marginal lands and under low supply of water, thus a crop of the future [24]. However, cowpea production under water deficit reduce yields to 360 kg / ha especially during pre-flowering when compared to 1000 kg / ha under adequate water supply, meaning that cowpea is significantly impinged by recurrent drought and a long duration of drought [31, 1, 4, 22]. Additionally, Hall [15] reported that cowpea is susceptible to severe water deficits specifically during pod setting and grain filling stages. Drought remains the single most important factor threatening the food security of peoples in the developing world.

The selection of drought-tolerant genotypes is one of the alternatives to minimize the effects of stress on yield [4, 20]. Exploration of genetic diversity, knowledge of the nature and extent of action of genes controlling quantitative traits proves to be necessary for the successful development of crop varieties through appropriate choice of parents for breeding programs [10, 14]. Indeed, selection of parents is one of the key steps in breeding programs involving crosses, since the success of a breeding program is associated with the segregation potential of the populations generated by the crosses, which is the combining abilities of the parental genotypes used. Diallel analyses aim at evaluating genetic design, estimating useful parameters in the selection of parents for crosses and explaining the genetic effects involved in determining their characteristics. To this end, Griffing [14] proposed the most commonly used methods, which estimates the general combining ability (GCA) and the specific combining ability (SCA) of genotypes resulting from diallel crosses [28]. Combining ability connotes the hybridization value of the parental genotypes involved in the crossing to develop hybrids [32]. The best parents are selected through combining ability methods and subsequently used to produce high yielding and resistant hybrids. The combining ability was first applied in maize, then efficaciously to other crops. The general combining ability determines the potentials of the parents in general on the principle of additive gene action while the specific combining ability effect indicates the potentials of the hybrid in specific terms based on the action of the dominant gene [29]. Better understanding and knowledge about the combination of gene ability and effects will be of use to cowpea breeders in selecting ideal parents, breeding strategies and identifying potential genotypes among segregating populations to improve cowpea productivity especially changing climate [24]. Diallel crosses therefore become indispensable approach for broadening the genetic constitution of cowpea and for developing superior segregating populations [28]. Thus, the objective of this work was to (i)identify cowpea parents and genotype combinations with a high probability of generating drought-tolerant segregating populations, and (ii) understand the nature and action of genes controlling traits directly related to water deficit tolerance.

## Materials and methods

### Experimental site and plant material

This study was carried out under a controlled environment at the International Institute of Tropical Agriculture (IITA-Benin). Twenty (20) genotypes were previously screened and 8 genotypes were identified as drought-resistant and drought-susceptible genotypes [9]. The hybridization of the 8 genotypes was performed using a half diallel (4 × 4) crossbreeding design and 28 F1 hybrids were developed out of which sixteen (16) hybrids (F1) were selected for drought stress evaluation based on the availability of greenhouse spaces (Table 1). This hybridization process followed 3 important steps: pollen collection, emasculation and pollination. The collection of pollens grains was performed during the floral opening of the cowpea flowers between 5 a.m. and 9 a.m. The collected grain pollens were then kept in a refrigerator for their use in the evening during pollination when the stigma was more receptive. Emasculation and pollination were carried out simultaneously. The success of pollination and fertilization was observed when pollination was performed around 7 p.m. In addition to the developed hybrids (F1), the 8 parents were also used for the evaluation of drought stress (Table 1). A total of 24 cowpea genotypes were subjected to water deficit conditions in a split plot design.

**Table 1:** Eight parents and 16 hybrids (F1) used for drought stress

Types	Descriptors	Genotypes	Response
	P1	KVX 61–1	Tolerant
	P2	IT06K242-3	Tolerant
	P3	IT07K-211–1-8	Tolerant
Parents	P4	Kpodjiguèguè	Tolerant
	P5	IT99K-573–1-1	Sensitive
	P6	Tawa	Sensitive
	P7	KVX 396–18	Sensitive
	P8	IT97K-206–1-1	Sensitive
	P1 x P5	KVX 61–1 × IT99K-573–1-1	-
	P1 x P6	KVX 61–1 × Tawa	-
	P1 x P7	KVX 61–1 × KVX 396–18	-
	P1 x P8	KVX 61–1 × IT97K-206–1-1	-
	P2 x P5	IT06K242-3 × IT99K-573–1-1	-
	P2 x P6	IT06K242-3 × Tawa	-
	P2 x P7	IT06K242-3 × KVX 396–18	-
Hybrids F1	P2 x P8	IT06K242-3 × IT97K-206–1-1	-
	P3 x P5	IT07K-211–1-8 × IT99K-573–1-1	-
	P3 x P6	IT07K-211–1-8 × Tawa	-
	P3 x P7	IT07K-211–1-8 × KVX 396–18	-
	P3 x P8	IT07K-211–1-8 × IT97K-206–1-1	-
	P4 x P5	Kpodjiguèguè x IT99K-573–1-1	-
	P4 x P6	Kpodjiguèguè x Tawa	-
	P4 x P7	Kpodjiguèguè x KVX 396–18	-
	P4 x P8	Kpodjiguèguè x IT97K-206–1-1	-

### Data collection

Morphological, physiological and agronomic data were collected on the plants during the study.

i. Number of pods per plant (NPP): average of mature pods of a plant
ii. Yield (YLD): Yield per pot weighted in grams (g)
iii. Hundred Seed Weight (g) (100SW): randomly count of 100 seeds from a plant and then weighed using a precision digital scale.
iv. Number of days to flowering (NDF): number of days from sowing to the stage when the plants had open flowers.
v. Seeds per pod (NSP): average seed count of 10 pods,
vi. Pod Length (cm) (PL): average of 10 randomly selected fully mature pods.
vii. Leaf length cm (Ll): the flag leaf was measured using a metric ruler.
viii. Leaf width cm (LW): it was measured using a metric ruler at the level of the flag leaf.
ix. Plant height (PH): was measured using a metric ruler from ground level at the base to the tip of the plant meristem expressed in cm.
x. Number of leaves per plant (NLP): total number of leaves present on the plant.
xi. Number of days to pod maturity (NDPM): recorded when pods reached physiological maturity
xii. Photosynthetic yield (Fv_Fm): it was measured using an Os30p + fluorometer. It is a machine that measures the maximum quantum efficiency of PSII in plants. Noted as Fv/Fm ratio, with Fv = Fm-Fo. With Fv: variable fluorescence, Fm: maximum fluorescence and Fo: minimum fluorescence. This ratio shows a high degree of correlation with the quantum efficiency of net photosynthesis. Generally, in a healthy, unstressed plant, this photosynthetic yield is around 0.800 and in the event of stress this value decreases.
xiii. Photosynthetic efficiency (Fv_Fo): measured using an Os30p + fluorometer. Here, it allows the detection of the presence of stress. In a healthy, unstressed plant, the photosynthetic efficiency is about 2.5 to 4 and under stress this value decreases.
xiv. Leaf chlorophyll content (LCC): The SPAD Meter was used to measure the chlorophyll content at 30, 45 and 60 DAP.
xv. Leaf proline content (LPC): a proline assay protocol was used for the proline content of the plants.

#### Proline assay protocol

Proline, an amino acid whose content increases rapidly in plants under water deficit, is suggested as an evaluation parameter for irrigation planning and selection of drought-resistant varieties. Thus for its dosage, it is necessary to collect fresh tissue (0.25 g) which is homogenized in 10 ml of water with 3% aqueous sulfosalicylic acid. After 3 h the mixture is centrifuged at 1500 revolutions for 10 min. It is then necessary to take 2 ml of the supernatant and add 2 ml of glacial acetic acid and 2 ml of hydronin acid. The whole is boiled at 100˚C in a sea bath for one hour. The reaction is stopped by placing it on ice. Four (04) ml of Toluene is added and mixed vigorously using the vortex for 15 to 20 s and the toluene containing the chromophore was separated using a separating funnel and the absorbance was measured at 520 nm in a spectrophotometer against an appropriate blank which is toluene. The proline content was determined from a standard curve prepared with pure proline and expressed in mg/g. MS. The unknown proline content is calculated from the samples using the standard graph. The proline content is then calculated with the formula:

$$Tproline=\frac{e\, x V1 x V2}{Weight\, FM \,x \,115.5}$$

With :

e= μg/ml proline

V1= volume of toluene

V2= volume of sulfosalicylic acid

Weight FM= the gram of fresh matter

### Statistical analysis

Statistical analysis was performed with RStudio software 1.3.1093. The lmDiallel package was used to calculate combining abilities according to method 2 of Griffing (1986) where parents and F1 hybrids are included. F1 reciprocity was excluded in this method. The estimation of genetic parameters was obtained using the variability package. The linear model of aptitude for the combination for the half diallel considering (p (*p* + 1)/2 is written: ${\varvec{x}}{\varvec{i}}{\varvec{j}}={\varvec{\mu}}+{{\varvec{g}}{\varvec{c}}{\varvec{a}}}_{{\varvec{i}}}+{{\varvec{g}}{\varvec{c}}{\varvec{a}}}_{{\varvec{j}}}+{{\varvec{s}}{\varvec{c}}{\varvec{a}}}_{{\varvec{i}}{\varvec{j}}}+{\varvec{e}}{\varvec{i}}{\varvec{j}}$ for the observations of the crossing *i* × *j*.

where: *i* = 1, 2,…. ….*p*

μ = population general mean

*gca*_*i*_ = general combining ability of the *i* ^th^ line or variety

*sca*_*ij*_ = specific combining ability of the crossing *i* × *j*

e_ij_ = error associated with the observations of the ij crossing

General combining ability for males and females was calculated as: $\boldsymbol G\boldsymbol C{\boldsymbol A}_{\boldsymbol f}\boldsymbol={\boldsymbol X}_{\boldsymbol f}\boldsymbol-\boldsymbol\mu\boldsymbol\;\boldsymbol a\boldsymbol n\boldsymbol d\boldsymbol\;\boldsymbol G\boldsymbol C{\boldsymbol A}_{\boldsymbol m}\boldsymbol={\boldsymbol X}_{\boldsymbol m}\boldsymbol-\boldsymbol\mu$  

where:

X_f_ and X_m_ = Average of female and male parents, respectively

GCA_m_ and GCA_f_ = general combining ability of male and female parents, respectively

μ = overall average of crosses in the trial.

Specific combining ability was calculated using the proposed formula:

$${\mathbf{SCA}}_{\mathbf X}\boldsymbol={\mathbf X}_{\mathbf x}\boldsymbol-\mathbf E\left({\mathbf X}_{\mathbf x}\right)={\mathbf X}_{\mathbf x}-\left[{\mathbf{GCA}}_{\mathbf{xf}}\boldsymbol+{\mathbf{GCA}}_{\mathbf m}\boldsymbol+\mathbf\mu\right]$$

where:

X_x_ = average value observed in the crossing

E (X_x_) = Expected average value of the cross based on the 2 GCAs of its parents

SCAx = specific combining ability of cross x

The coefficient of heritability in the broad sense (H^2^) was estimated by the formula:

$$\left(H^{2}\right)=\frac{\mathbf\sigma^{\mathbf2}\boldsymbol G\boldsymbol C\boldsymbol A\boldsymbol f\boldsymbol+\mathbf\sigma^{\mathbf2}\boldsymbol G\boldsymbol C\boldsymbol A\boldsymbol m\boldsymbol+\mathbf\sigma^{\mathbf2}\boldsymbol S\boldsymbol C\boldsymbol A\boldsymbol f}{\boldsymbol\sigma^{\mathbf2}\boldsymbol G\boldsymbol C\boldsymbol A\boldsymbol f\boldsymbol+\boldsymbol\sigma^{\mathbf2}\boldsymbol G\boldsymbol C\boldsymbol A\boldsymbol m\boldsymbol+\mathbf\sigma^{\mathbf2}\boldsymbol S\boldsymbol C\boldsymbol A\boldsymbol m\boldsymbol+\mathbf\sigma^{\mathbf2}\boldsymbol e}$$

Where:

${{\varvec{\sigma}}}^{2}{\varvec{G}}{\varvec{C}}{\varvec{A}}{\varvec{f}}$= general combining ability of female variance

${{\varvec{\sigma}}}^{2}{\varvec{G}}{\varvec{C}}{\varvec{A}}{\varvec{m}}$=general combining ability of male variance

${{\varvec{\sigma}}}^{2}{\varvec{S}}{\varvec{C}}{\varvec{A}}{\varvec{f}}$ =Specific combining ability of female variance

${{\varvec{\upsigma}}}^{2}{\varvec{S}}{\varvec{C}}{\varvec{A}}{\varvec{m}}$= Specific combining ability of male variance

$${{\varvec{\upsigma}}}^{2}{\varvec{e}}$$=error variance

## Results

### Estimating the effects of specific and general combining ability

There were significant differences between the mean squares for the combining ability for the fifteen variables studied (Table 2). Under normal irrigation condition, General Combining Ability (GCA) analysis showed significant differences (*p* < 0.001) for plant height, number of leaves per plant, leaf chlorophyll content, proline content, number of days to flowering, number of days to pod maturity and hundred-seed weight. Under water deficit conditions, only plant height, chlorophyll content, proline content, number of days to flowering, number of days to pod maturity days and hundred-seed weight showed highly significant differences ( *p* < 0.001). Significant differences were recorded in the analysis of specific combining ability (SCA). Under normal irrigation condition, analysis of specific combining ability showed that the mean square was significant for plant height, number of leaves per plant, chlorophyll content, number days to flowering, number days to pod maturity and hundred-seed weight. Under water deficit condition, SCA showed that the mean square was not significant for all traits except plant height, number of flowering days, proline content and number of days to maturity of the pods. Highly significant ANOVA estimates of specific and general combining abilities indicate the importance of additive and non-additive genes in the expression of these traits.

**Table 2:** Values of the half-diallel mean squares of cowpea genotypes under conditions of water deficit and normal irrigation

	Mean Square					
Parameters	GCA		SCA		GCA/SCA	
	R0	R1	R0	R1	R0	R1
PH	2769.81***	2462.46***	2506.33***	2379.77***	2638,07	2428,115
LW	8.1901	8.8212	8.1361	8.8247	8,1631	8,8229
Ll	12.989	15.302	13.101	15.281	13,045	15,2915
NLP	61.426**	35.113	57.538***	36.039*	59,482	35,576
LCC	748.63***	1043.22***	760.69***	1013.52	754,66	1028,37
Fv_Fm	0.2467	0.1360	0.2426	0.1342	0,24465	0,1351
Fv_Fo	2.1390	2.6964	2.1134	2.6312	2,1262	2,6638
LPC	1973.75***	1348.37***	1785.80	1184.45***	1879,775	1266,41
NDF	433.46***	556.49***	443.99***	550.34	438,725	553,415
NDPM	885.19***	954.70***	892.99***	949.78***	889,09	952,24
NPP	10.235	7.1539	10.572	7.0541	10,4035	7,104
PL	30.619	25.591	32.011*	24.643	31,315	25,117
NSP	10.592	7.8737	10.875	7.7979	10,7335	7,8358
100SW	6923.0***	863.185**	7026.3***	861.414***	6974,65	862,2995
YLD	9.1266	5.3955	8.9510	6.0247	9,0388	5,7101

*GCA* General combining ability, *SCA* Specific combining ability, *GCA/SCA* ratio General combining ability and Specific combining ability, *PH* plant height, *LW* leaf width, *Ll* leaf length, *NLP* number of leaves per plant, *LCC* leaf chlorophyll content, *Fv_Fm* photosynthetic yield, *Fv_Fo* photosynthetic efficiency, *LPC* leaf proline content, *NDF* number of days to flowering, *NDPM* number of days to pod maturity, *NPP* number of pods per plant, *PL* Pod length, *NSP* number of seed per pod, *100SW* weight of one hundred seeds, *YLD* seed yield, *R0* water regime (normal irrigation), *R1* water regime (water deficit condition)

^*^: significant (p < 0.05)

^**^: significant (p < 0.01)

^***^ significant (p < 0.001)

### Effect of general combining ability of the parents

The estimation of the general combining ability varied among the eight parental genotypes for the four morphological traits and are presented in Table 3. Under normal and drought conditions, significant and positive GCA effects for plant height were observed for all the parents except P7, which recorded significant but negative GCA effects. KVX396-18 (P7) was a good progenitor for plant height under the two imposed water regime conditions in this study (Table 3). The genotypes KVX61-1 (P1), IT99K-573–1-1 (P5) and Tawa (P6) were found to be better progenitors for the number of leaves per plant (NLP) under normal irrigation conditions where they showed a highly significant general combining ability (GCA) effect. KVX61-1 (P1), IT06K242-3 (P2), IT07K-211–1- 8 (P3), Kpodjiguèguè (P4), IT99K-573–1-1 (P5), Tawa (P6) and IT97K-206–1-1 (P8) demonstrated significantly positive and very high general combining ability effects for plant height under both water regime conditions. Under normal irrigation conditions, parents P1, P5 and P6 exhibited a significant positive GCA effect for the number of leaves per plant (NLP). As for the chlorophyll content and proline content, under the two water regime conditions, KVX61-1, IT06K242-3, IT07K-211–1-8, Kpodjiguèguè, IT99K-573–1-1, Tawa and IT97K-206 -1–1 recorded relatively positive and significant GCA effects while KVX396-18 had a significant and negative GCA effect (Table 4). Under both water regimes, P1, P2, P3, P4, P5, P6 and P8 were significant and positive GCA effects for the number of days to flowering (NDF) and number of days to pod maturity (NDPM) while P7 had a negative and high GCA effect (Table 5). Under normal irrigation condition, P1, P3, P4 and P5 showed significant and negative GCA effects for hundred seed weight while P2 and P6 recorded relatively highly significant and positive GCA effects. However, under water deficit conditions, P1, P2, P3, P5 and P8 had positive and significant GCA effects for hundred seed weight trait.

**Table 3:** Combining ability (GCA) of 8 cowpea parents involving half-diallel analysis for morphological traits under water deficit and normal irrigation conditions

Parents	PH		LW		Ll		NLP	
	R0	R1	R0	R1	R0	R1	R0	R1
P1	41.35***	37.36***	2.505	2.616	2.859	3.744	7.086	4.864
P2	39.48***	36.34***	2.366	2.459	3.180	3.361	5.982*	4.778
P3	39.71***	36.87***	2.281	2.500	2.837	3.139	5.186	4.693
P4	57.90***	47.15***	2.546	2.343	3.485	3.758	7.405	4.645
P5	32.64***	32.13***	2.185	2.307	2.635	3.037	5.362*	4.773
P6	53.34***	50.63***	2.743	2.833	3.540	3.742	7.620*	5.421
P7	-255.32***	-228.22***	-13.973	-14.328	-17.769	-20.047	-36.945	-27.551
P8	246.20***	215.92***	13.317	13.594	17.000	19.311	35.246	25.924

^*^: significant (p < 0.5)

^***^: significant (p < 0.001)

**Table 4:** General combining ability (GCA) of 8 cowpea parents involving half-diallel analysis for physiological traits under water deficit and normal irrigation conditions

Parents	Fv_Fm		Fv_Fo		LCC		LPC	
	R0	R1	R0	R1	R0	R1	R0	R1
P1	0.309	0.316	1.137	1.411	25.63***	20.70***	36.19***	28.69***
P2	0.618	0.352	1.440	1.560	22.58***	29.13***	39.97***	28.83***
P3	0.337	0.337	1.215	1.598	22.68***	26.73***	18.72***	31.55***
P4	0.374	0.352	1.324	1.642	23.34***	22.89***	21.68***	44.74***
P5	0.319	0.294	1.206	1.255	24.62***	31.99***	18.74***	32.71***
P6	0.392	0.352	1.341	1.596	23.21***	21.59***	44.04***	26.11***
P7	-2.318	-1.941	-7.353	-8.835	-135.73***	-144.43***	-169.26***	-189.35***
P8	2.283	1.877	7.040	8.606	129.40***	135.81***	159.16***	186.04***

^***^: significant (p < 0.001)

**Table 5:** Effects of general combining ability (GCA) of 8 cowpea parents involving half-diallel analysis for agronomic traits under water deficit and normal irrigation conditions

Parents	NDF		NDPM		NPP		PL		NSP		100SW		YLD	
	R0	R1	R0	R1	R0	R1	R0	R1	R0	R1	R0	R1	R0	R1
P1	18.61***	23.73***	24.91***	29.00***	3.132	1.984	5.134	3.852	3.160	1.860	-16.76***	6.51*	2.540	1.603
P2	17.00***	17.40***	23.86***	24.19***	2.751	2.650	4.719	4.514	2.850	2.068	53.22***	9.18**	2.200	1.738
P3	19.04***	20.87***	28.10***	27.28***	2.703	2.031	4.424	5.313	2.626	2.708	-16.58***	6.78*	2.659	1.758
P4	19.42**	18.87***	26.86***	26.00***	2.894	1.555	5.540	4.920	3.365	2.586	-17.38***	6.06	2.827	1.872
P5	17.428***	21.54***	26.33***	28.28***	2.084	2.269	4.748	3.610	2.665	2.400	-16.92***	7.42*	1.971	2.308
P6	19.14***	19.11***	25.81***	25.57***	3.322	2.174	4.918	4.971	2.984	2.381	52.86***	5.80	3.107	1.579
P7	-106.33***	-116.36***	-149.22***	-153.29***	-16.37	-11.944	-28.39	-26.469	-17.121	-13.323	-16.58	-40.66	-14.690	-10.174
P8	102.00***	111.17***	142.56***	146.25***	15.870	11.22	27.31	25.755	16.591	12.640	-5.25	39.53*	14.073	9.488

^*^: significant (p < 0.05)

^**^: significant (p < 0.01)

^***^ significant (p < 0.001)

### Effects of specific combining ability

Estimates of SCA effects of cowpea hybrids for morphological traits are presented in Table 6. The SCA effects of the hybrids obtained from the crosses for the traits showed a very wide variation KVX61-1 × IT97K-206–1-1 (P1 x P8), IT06K342-3 × Tawa (P2 x P6), IT06K342-3 × IT97K-206–1-1 (P2 x P8), IT07K-211–1-8 × IT99K-573–1-1 (P3 x P5) and Kpodjiguèguè x IT97K-206–1-1 (P4 x P8) showed negative and significant SCA effects for plant height under normal irrigation conditions. Under this same condition, the height of the plant had positive and highly significant SCA effects for the hybrids such as KVX61-1 × KVX396-18 (P1 x P7), IT06K342-3 × KVX396-18 (P2 x P7), IT07K-211- 1–8 × KVX396-18 (P3 x P7) and Kpodjiguèguè x KVX396-18 (P4 x P7). Under water deficit conditions, crosses P1 x P6, P1 x P7, P2 x P7, P3 x P5, P3 x P7 and P4 x P7 showed positive and significant SCA effects for plant height while P1 x P5, P1 x P8, P2 x P5, P2 x P6, P2 x P8, P3 x P8 and P4 x P8 recorded negative and highly significant SCA effects. Under normal water regime conditions, crosses P1 x P7, P2 x P7, P3 x P7 and P4 x P7 exhibited positive and significant SCA effects for number of leaves per plant (NLP). Crosses P1 x P7, P2 x P7, P3 x P7 and P4 x P7 showed positive and highly significant SCA effects for chlorophyll content and proline content under both water regime conditions. However, crosses P1 x P8, P2 x P8, P3 x P8 and P4 x P8 had negative and significant SCA effects for chlorophyll content and proline content under both water regime conditions (Table 7). In addition, P1 x P5 exhibited a positive and significant SCA effect for chlorophyll content while crosses P1 x P6 and P2 x P6 had positive and significant SCA effect for proline content under normal irrigation conditions. Under the same conditions, P2 x P5, P3 x P6 and P4 x P6 recorded negative and significant SCA effects for proline content. Under water deficit condition, P1 x P6 had negative and significant SCA effect for chlorophyll content and proline content. The crosses P2 x P5 and P4 x P5 demonstrated positive and significant SCA effect for chlorophyll content under water deficit conditions. The number of days to flowering and number of days to pod maturity expressed significant and positive SCA effects by all the hybrids under the two water regime conditions except P1 x P5, P1 x P6, P1 x P8, P2 x P5, P2 x P6, P2 x P8, P3 x P5, P3 x P8, P4 x P5, P4 x P6 and P4 x P8 (Table 8). In contrast, under both regime conditions, P1 x P8, P2 x P8, P3 x P8, and P4 x P8 had relatively high-negative and significant SCA effects for NDF and NDPM. For seed yield (YLD) and number of seeds per pod (NSP), P1 x P8, P2 x P5, P2 x P8, P3 x P8 and P4 x P8 expressed negative and relatively poor SCA effects under both water regime conditions (Table 9). The best specific combiners for YLD were P1 x P7, P2 x P7, P3 x P7 and P4 x P7 under both water regime conditions. Under normal irrigation conditions, 100SW expressed positive and significant SCA effects by P1 x P5, P2 x P6 and P4 x P5, which were the best combiners. Negative and significant SCA effects are recorded by P1 x P6, P2 x P5, P2 x P7, P2 x P8, P3 x P6 and P4 x P6 for 100SW. Under drought condition, P1 x P7, P2 x P7, P3 x P7 and P4 x P7 exhibited positive and significant SCA effects for 100SW, thus they were the best combiners.

**Table 6:** Effects of specific combining ability (SCA) of cowpea hybrids involving half-diallel analysis for morphological traits under conditions of water deficit and normal irrigation

Hybrids	PH		LW		Ll		NLP	
	R0	R1	R0	R1	R0	R1	R0	R1
P1xP5	-3.11	-3.46	-0.11	0.17	-0.397	-0.651	0.384	2.498
P1xP6	1.47	21.19***	-1.18	0.08	0.664	0.244	3.027	-1.579
P1xP7	276.34***	246.79***	16.74	16.81	20.208	23.91	42.72*	31.02
P1xP8	-191.01***	-173.69***	-9.57	-10.69	-14.229	-15.37	-29.36	-19.41
P2xP5	-3.28	-10.78*	0.23	-0.33	-1.885	-1.101	-3.577	-0.079
P2xP6	-12.72*	-16.90**	0.68	0.67	0.660	-0.65	1.198	1.672
P2xP7	292.59***	270.14***	14.95	15.58	21.32	24.18	46.09*	30.34
P2xP8	-198.47***	-154.70***	-10.42	-10.02	-12.88	-15.44	-30.26	-19.66
P3xP5	-27.96***	-5.97	-0.58	-0.12	1.524	-0.763	-0.08	-0.760
P3xP6	1.515	31.13***	0.78	-0.46	-1.480	-0.717	0.827	5.258
P3xP7	308.15***	260.71***	15.50	16.92	18.529	21.43	44.95*	30.26
P3xP8	-202.92***	-196.54***	-10.50	-10.31	-12.39	-13.63	-34.63	-22.74
P4xP5	-9.67	8.07	-0.05	0.21	-0.623	0.567	0.498	0.187
P4xP6	27.80***	7.92	0.87	1.22	1.488	1.296	-1.058	-1.56
P4xP7	310.52***	278.55***	16.12	16.26	21.498	22.93	47.20*	32.94
P4xP8	-195.28***	-174.38***	-10.94	-12.15	-14.23	-16.62	-28.91	-19.69

^*^: significant (p < 0.05)

^**^: significant (p < 0.01)

^***^: significant (p < 0.001)

**Table 7:** Effects of specific combining ability (SCA) of cowpea hybrids involving half-diallel analysis for physiological traits under water deficit and normal irrigation conditions

Hybrids	Fv_Fm		Fv_Fo		LCC		LPC	
	R0	R1	R0	R1	R0	R1	R0	R1
P1xP5	-0.135	-0.114	-0.139	-0.491	10.24**	-2.319	5.566	-6.39
P1xP6	-0.084	0.020	-0.233	-0.130	0.338	-9.60*	58.54***	-12.09*
P1xP7	2.692	2.274	8.819	10.389	154.63***	176.74***	188.66***	219.84***
P1xP8	-2.082	-1.556	-6.017	-7.239	-104.47***	-113.17***	-156.48***	-157.54***
P2xP5	-0.298	-0.070	0.253	-0.978	7.696	19.24***	-36.47***	-6.197
P2xP6	-0.282	0.027	0.171	0.126	0.1395238	3.889	36.87***	-10.03
P2xP7	2.410	2.330	8.584	10.70	155.10***	165.28***	258.40***	206.97***
P2xP8	-0.510	-1.554	-5.669	-6.876	-111.35***	-111.47***	-151.19***	-146.51***
P3xP5	-0.083	-0.068	-0.118	-0.076	-8.120	3.932	8.576	-13.38**
P3xP6	0.099	-0.025	0.173	-0.521	4.539	-2.005	-27.75***	-9.990
P3xP7	2.431	2.276	8.299	10.424	170.28***	193.74***	188.44***	227.81***
P3xP8	-1.971	-1.495	-5.690	-6.739	-114.81***	-125.93***	-125.38***	-152.03***
P4xP5	-0.025	0.033	-0.240	0.0143	-4.86	10.14*	2.375	3.553
P4xP6	-0.055	-0.067	0.046	0.016	-4.286	7.544	-11.71*	-10.10
P4xP7	2.668	2.280	8.918	10.162	167.30***	152.06***	206.19***	211.24***
P4xP8	-2.002	-1.514	-5.733	-6.971	-104.29***	-111.51***	-144.09***	-112.72***

^*^: significant (p < 0.05)

^**^: significant (p < 0.01)

^***^: significant (p < 0.001)

**Table 8:** Effects of specific combining ability (SCA) of cowpea hybrids involving half-diallel analysis for agronomic traits: Number of Days to Flower (NDF), Number of Days to Pod Maturity (NDPM), Number of Pods per Plant (NPP), pod length (¨PL) under water deficit and normal irrigation conditions

Hybrids	NDF		NDPM		NPP		PL	
	R0	R1	R0	R1	R0	R1	R0	R1
P1xP5	-6.714	8.529	-5.244	7.339	-0.392	0.023	-0.33	-2.754
P1xP6	7.238	-2.375	6.613	-1.613	1.7023	-0.214	0.706	1.084
P1xP7	121.04***	138.10***	166.98***	180.91***	21.404	14.90	33.08	31.53
P1xP8	-80.61***	-87.76***	-112.47***	-118.29***	-16.178	-9.59	-22.28	-3.3225
P2xP5	-3.095	-7.13	1.136	-3.517	-1.678	1.023	-3.322	-0.005
P2xP6	-5.47	-4.041	-5.672	-2.470	1.083	0.785	1.396	-0.960
P2xP7	132.33***	134.77***	178.70***	178.39***	17.787	15.904	33.99	31.161
P2xP8	-87.66***	-86.10***	-121.42***	-118.48***	-11.797	-10.595	-22.14	-1.664
P3xP5	9.190	-1.27	7.232	-0.613	-0.964	-0.023	1.909	-0.408
P3xP6	0.476	3.8154762	5.422	1.101	-2.202	-0.261	-3.896	-0.265
P3xP7	120.28***	145.63***	175.13***	192.96***	20.500	14.52	30.454	29.64
P3xP8	-87.71***	-100.24***	-122.32***	-130.24***	-12.08	-8.976	-19.424	-18.041
P4xP5	-3.857	4.386	-1.196	3.339	-0.154	0.452	0.523	-1.744
P4xP6	-1.571	-0.184	-6.005	1.386	-0.059	0.880	1.076	-0.689
P4xP7	129.23***	135.29***	177.70***	179.25***	19.309	11.66	34.80	31.463
P4xP8	-80.42***	-97.57***	-108.75***	-124.62***	-13.27	-9.16	-24.02	-19.281

^*^: significant (p < 0.05)

^**^: significant (p < 0.01)

^***^: significant (p < 0.001)

**Table 9:** Effects of specific combining ability (SCA) of cowpea hybrids involving half-diallel analysis for agronomic traits: number of seeds per pod (NSP), hundred-seed weight (100SW), seed yield (YLD) under water deficit and normal irrigation conditions

Hybrids	NSP		100SW		YLD	
	R0	R1	R0	R1	R0	R1
P1xP5	-1.056	-0.809	27.14***	3.724	0.503	0.067
P1xP6	0.191	0.075	-45.7***	-5.55	1.216	-1.30
P1xP7	21.29	15.863	25.23	49.28*	16.14	13.30
P1xP8	-13.94	-0.916	16.38	-35.48	-12.23	-7.717
P2xP5	-0.812	-0.824	-46.18***	-0.31	-2.079	-0.20
P2xP6	0.335	-0.087	378.39***	1.872	0.918	1.495
P2xP7	20.02	15.438	-44.59*	45.77*	16.25	11.22
P2xP8	-3.995	-9.691	-54.66**	-27.36	-10.47	-7.76
P3xP5	-0.255	1.642	26.822***	-4.018	-1.350	-0.60
P3xP6	-1.374	0.447	-45.77***	-3.678	-0.443	-0.123
P3xP7	18.294	14.19	25.62	48.54*	18.21	12.96
P3xP8	-1.781	-9.531	16.85	-28.07	-10.43	-7.41
P4xP5	0.239	0.697	26.36***	-1.159	1.083	3.432
P4xP6	-0.080	0.215	-43.34***	0.720	-0.125	0.439
P4xP7	22.29	16.38	24.07	44.35*	16.78	9.034
P4xP8	-5.353	-0.909	14.02	-33.29	-11.24	-7.74

^*^: significant (p < 0.05)

^**^: significant (p < 0.01)

^***^ significant (p < 0.001)

### Estimation of genetic parameters

There was wide variation in the estimation of genetic parameters (Table 10). Thus, genotypic variance ranged from 0.0107 to 1185.3778 while phenotypic variance varied from 0.0612 to 7596.1417. The highest genotypic and phenotypic variances were obtained for proline content (LPC), plant height (PH), number of leaves per plant (NLP), number of days of pod maturity (NDPM), number of pods per plant (NPP), pod length (PL) and hundred seed weight (100SW). Leaf width (LW) (10.9733), chlorophyll content (LCC) (14.4015), number of flowering days (NDF) (13.4262), seed yield (YLD) (7.6032) photosynthetic yield (Fv_Fm) (0.0107), photosynthetic efficiency (Fv_Fo) (1.2524) and number of seeds per pod (NSP) (0.3956) recorded low genotypic variance (GV < 20%). Medium values ​​with respect to phenotypic variance were observed in leaf width (28.0837), number of seeds per pod (23.5519) and seed yield (27.4199) traits while low values ​​are recorded for leaf length (3.2697), photosynthetic yield (0.0612) and photosynthetic efficiency (2.5872).

**Table 10:** Estimation of the genetic parameters for the different variables studied

	GV	PV	(H^2^)	GM	GVC	PVC	GA	GA (%)
PH	430.2160	1047.2594	0.4108	98.2853	21.1035	32.9260	27.3859	27.8637
LW	10.9733	28.0837	0.1527	5.486	10.9733	28.0837	0.4846	8.8334
Ll	0.1935	3.2697	0.0592	8.6224	5.1017	20.9713	0.2204	2.5561
NLP	96.0496	504.4079	0.1904	24.1026	40.6616	93.1811	8.8099	36.5517
LCC	14.4015	629.9076	0.0229	50.1987	7.5598	49.9972	1.1821	2.3548
Fv_Fm	0.0107	0.0612	0.1748	0.7502	13.7889	32.9772	0.0891	11.8773
Fv_Fo	1.2524	2.6872	0.4661	3.7538	29.8126	43.6694	1.5738	41.9254
LPC	1185.3778	7596.1417	0.156	86.5365	39.7859	100.7158	28.0174	32.3764
NDF	13.4262	193.7365	0.0693	46.5385	7.8734	29.9084	1.9871	4.2698
NDPM	405.2426	761.1729	0.5324	79.4744	25.3297	34.7148	30.2580	38.0727
NPP	770.1795	984.5872	0.7822	31.6795	87.6028	99.0487	50.5629	159.6077
PL	27.3551	47.6634	0.5739	13.5331	38.6475	51.0147	8.1623	60.3136
NSP	0.3956	23.5519	0.0168	8.39	7.4966	57.843	0.1679	2.0012
100SW	953.9867	1125.2180	0.8478	35.3837	87.2906	94.8014	58.5856	165.5722
YLD	7.6032	27.4199	0.2773	5.9528	46.3209	87.9654	2.9911	50.247

*GV* Genotypic Variance, *PV* Phenotypic Variance, (*H* ^*2*^) Broad Sense Heritability, *GM* Overall Trait Mean, *GVC* Genotypic Variance Coefficient, *PVC* Phenotypic Variance Coefficient, *GA* Genetic Gain, *GA ( %)* Percentage of genetic gain

Very high genotypic and phenotypic coefficients of variation were observed in several traits (Table 10). Indeed, the estimate of the phenotypic coefficient of variation was higher than the estimate of the genotypic coefficient of variation in all traits studied. The highest genotypic coefficient of variation was observed for the number of pods per plant (87.6028) and hundred-seed weight (87.2906). The lowest coefficients were observed for leaf length (5.1017), LCC (7.5598), NDF (7.8734) and NSP (7.4966). The highest phenotypic coefficients of variation were observed for number of leaves per plant (93.1811), proline content (100.7158), number of pods per plant (99.0487), hundred-seed weight (94.8014) and seed yield (87.9654). However, the lowest coefficients were recorded by leaf width (28.0837), leaf length (20.9713) and number of days to flowering (29.9084). NDPM (0.5324), PL (0.5739), NPP (0.7822) and 100SW (0.8478) had the highest values ​​for heritability. Average values ​​were observed for PH (0.4108), photosynthetic efficiency (0.4661), leaf width (0.1527), number of leaves per plant (0.1904), photosynthetic yield (0.1748), proline content (0.156) and yield in seeds (0.2773). The lowest values ​​for heritability were recorded for leaf length (0.0592), chlorophyll content (0.0229), NDF (0.0693) and number of seeds per pod (0.0168).

The expected genetic gain in percent (GA %) varied significantly and ranged from 2.0012% to 165.5772%. The pod length (60.3136%), seed yield (50.247%), hundred-seed weight (165.5722%) and number of pods per plant (159.6077%) demonstrated the highest values for genetic gain. Average values ​​were recorded for plant height (27.8637%), number of leaves per plant (36.5517%), photosynthetic efficiency (41.9254%), proline content (32.3764%) and number of days of pod maturity (38.0727). %). The lowest values were observed with leaf length (2.5561%), chlorophyll content (2.3548%) and number of seeds per pod (2.0012%).

## Discussion

According to Griffing [14] and Ferreira et al. [12], significant GCA and SCA mean squares for the traits studied specify the importance of additive and non-additive gene effects for heritability. The results from this study showed that the mean squares for GCA and SCA are significant for the traits studied. Our results corroborate with those obtained by Owusu et al. [24] which revealed the presence of significant mean squares of GCA and SCA for traits such as YLD, NPP, NSP, 100SW, NDPM, plant height at flowering thus indicating the importance of genetic effects additive and non-additive in determination of these traits. Similar results were reported on cowpea by Ayo-Vaughan and Alake [2] for seed yield, number of pods per plant and weight of 100 seeds; and Pandey and Singh [25] for plant height (PH), number of pods per plant (NPP) and yield (YLD). Predominance of GCA effects over SCA effects for traits under water deficit conditions: plant height, chlorophyll content, proline content, number of flowering days, pod length, number of pods per plant, number of seed per pod, number of days of pods maturity and weight of hundred seeds show that the additive gene action is more important in the heritability of these traits. These results are similar to those obtained by Ayo-Vaughan and Alake [2] who observed significant effects of GCA on SCA for NPP and NSP in cowpea. Also, under water deficit conditions in our study, predominance of SCA effects over GCA effects was observed for number of leaves per plant and grain yield. This suggests that the action of non-additive genes is more important in transmission of these traits. In non-additive gene action, there is dominance traits and no aggregation of gene effects contrarily to the action of additive genes where the genes involved contribute to epistatic effects. The non-additive gene action calls for dominance gene action. This result corroborates with the results of Owusu et al. [24] who showed a predominance of SCA effects over GCA effects for YLD in cowpea. However, our results are not in line with those obtained by Romanus et al. [29] on cowpea who reported a predominance of GCA effects over SCA effects for yield and its components. However, differences between both results could be attributed to differences in the genetic material used as well as the research conditions (environment) under which studies were conducted. In the present study, under the two water regime conditions, all the parental genotypes studied seem to be good general combiners for plant height trait. In the case where the selection aims to reduce the height of the plants to allow plants to focus more on seed yield performance than on height performance, KVX396-18 (P7) genotype proves to be the ideal parent. Under normal irrigation conditions, P2, P5 and P6 are good progenitors to increase the number of leaves in plants. It is important to note that these genotypes are prioritized by cattle, sheep and goat breeders for the foliage supply of their subjects. This study allowed us to see that the genotypes P1, P2, P3, P4, P5, P6 and P8 have positive and significant general combining ability effects for chlorophyll content and proline content. This implies that these parental genotypes are good progenitors for increasing chlorophyll and proline content in plants and are also good combiners for improving the ability of cowpea to tolerate water deficit. Breeding programs aiming to improve drought tolerance in cowpea genotypes can then appropriate these progenitors in their research. These results are in line with those obtained on wheat by Hannachi et al. [16] who observed positive and significant GCA effects for the Waha and Ofanto genotypes on the chlorophyll content. These genotypes are therefore good progenitors for increasing chlorophyll production. Estimates of the general combining ability (GCA) effects of the eight cowpea genotypes subjected to the two water regime conditions showed that parents P1, P2, P3, P4, P5, P6 and P8 combined best NDF and NDPM. On the other hand, P7 combined better to reduce the number of days to flowering and the number of days of pod maturity. P7 is therefore the best genotype to use in breeding programs to obtain early varieties. Our results are in agreement with those provided by Romanus et al. [29] who showed positive and significant GCA effects of IT86D-716, IT87D-697–2, IT88D-867–11, IT86D-716 and IT92KD-405–1 for NDF. However, under normal irrigation conditions, KVX61-1 (P1), IT07K211-1–8 (P3), Kpodjiguèguè (P4), IT99K-573–1-1 (P5) and IT97K-206–1-1 (P8) were found to be poor general combiners for hundred-seed weight (100SW) while P2 and P6 were found to be good progenitors for this trait. Under water deficit conditions, P1, P2, P3, P5 and P8 show positive and significant GCA effects. This implies that these genotypes could be used in breeding programs to increase hundred-seed weight and hence seed yield. These results are similar to those obtained by Mwale et al. [21] on cowpea which showed positive and significant GCA effects for SECOW 5 T genotype on the hundred-seed weight trait under water deficit conditions. Specific combining ability (SCA) is considered a good criterion to select the best F2 populations for one or more desired traits [16]. In our study, KVX61-1 × Tawa (P1 x P6) and IT07K-211–1-8 × Tawa (P3 x P6) showed positive and significant SCA effects for plant height under water deficit conditions. KVX61-1 × KVX396-18 (P1 x P7), IT06K242-3 × KVX396-18 (P2 x P7), IT07K-211–1-1 × KVX396-18 (P3 x P7) and Kpodjiguèguè x KVX396-18 (P4 x P7) showed positive and significant specific combining ability (SCA) effects for plant height under both water regime conditions. This implies that these combinations performed better than predicted based on the GCA effects of their parents. Conversely, significant but negative SCA effects were obtained by hybrids KVX61-1 × IT97K-206–1-1 (P1 x P8), IT06K242-3 × IT97K-206–1-1 (P2 x P8), IT07K-211–1-1 × IT97K-206–1-1 (P3 x P8) and Kpodjiguèguè x IT97K-206–1-1 (P4 x P8). Hannachi et al. [16] found similar results showing negative and significant SCA effects for Waha x Ofanto for plant height. Under normal irrigation conditions, combinations KVX61-1 × KVX396-18 (P1 x P7), IT06K242-3 × KVX396-18 (P2 x P7), IT07K-211–1-1 × KVX396-18 (P3 x P7) and Kpodjiguèguè x KVX396-18 (P4 x P7) showed positive and significant SCA effects for number of leaf per plant. As such, crosses are expected to produce desirable segregants and could be exploited in cowpea breeding programs. High SCA effects and performance for LCC, LPC, NDF, NDPM and 100SW displayed by KVX61-1 × KVX396-18 (P1 x P7), IT06K242-3 × KVX396-18 (P2 x P7), IT07K-211–1-1 × KVX396-18 (P3 x P7) and Kpodjiguèguè x KVX396-18 (P4 x P7) further confirm the preponderance of non-additive gene action governing the inheritance of these traits. High SCA effects of KVX61-1 × KVX396-18 (P1 x P7), IT06K242-3 × KVX396-18 (P2 x P7), IT07K-211–1-1 × KVX396-18 (P3 x P7), Kpodjiguèguè x KVX396-18 (P4 x P7), KVX61-1 × IT97K-206–1-1 (P1 x P8), IT06K242-3 × IT97K-206–1-1 (P2 x P8), IT07K-211–1-1 × IT97K-206–1-1 (P3 x P8) and Kpodjiguèguè x IT97K-206–1-1 (P4 x P8) resulting from parents with high GCA values ​​each for LCC, LPC, NDF, NDPM and 100SW could be due to complementary of high combination loci. These hybrids could be exploited by a pedigree selection method to obtain transgressive segregants [3]. All crosses with significant SCA effects for the studied traits involved parents with high × high combining ability. According to Patij and Navale [26], such a result indicates presence of an allelic interaction in the expression of these traits. Similar results of high SCA values ​​for cowpea crosses were reported in previous studies by Ayo-Vaughan and Alake [2] for 100SW and by Pandy and Singh (2010) for PH and NDF.

According to Ibrahim [17], estimation of genetic parameters and the understanding of the mode of transmission of quantitative traits are essential in any crop improvement program. Analysis of the genetic parameters of all traits studied shows that the phenotypic variance is greater than genotypic variance. These results are consistent with those of Ezin et al. [8] on butternut squash and Dar and Sharma [7] on tomato. More or less small difference between phenotypic and genotypic variance for all traits would definitely reflect a poor impact of environmental factors on the expression of these traits [6]. The extent of total variability present in a trait is indicated by coefficient of variation [5]. In our study, the highest genotypic variation coefficient was observed for the number of pods per plant (87.6028) and hundred-seed weight (87.2906) traits. The lowest coefficients are observed for leaf length (5.1017), chlorophyll content (7.5598), number of days to flowering (7.8734) and number of seeds per pod (7.4966). The highest phenotypic coefficients of variation are observed for number of leaves per plant (93.1811), proline content (100.7158), number of pods per plant (99.0487), hundred-seed weight (94.8014) and grain yield (87.9654). However, the lowest coefficients were recorded by leaf width (28.0837), leaf length (20.9713) and number of days to flowering (29.9084). This means that leaf length, leaf width, number of days to flowering, chlorophyll content and number of seeds per pod do not explain existing genetic variability within these cowpea genotypes. Proline content, number of leaves per plant, number of pods per plant, hundred-seed weight and seed yield with a high phenotypic coefficient of variation show significant impact of environmental factors. Phenotypic coefficient of variation estimates was higher than genotypic coefficient of variation estimates for many traits. These results suggest that apparent variation is not only due to genotypes, but also to environmental influence Patij and Navale [26]. Similar results were reported by Patij and Navale [26],Torkadi et al. [35],Tomar et al. [34] on melon, Dar and Sharma [7],Singh and Singh [30] on tomato. It should be noted that small proportion of the difference between these two coefficients shows that these traits are very little influenced by the environment [18, 19]. According to Johnson et al. [18], a trait is highly heritable when the heritability is above 50%, poorly heritable when the heritability is below 20% and moderately heritable when the heritability is between 20 and 50%. In our study, heritability varied between 1.68% and 84.78%. Highly heritable traits are NDPM (53.24%), PL (57.39%), NPP (78.22%) and 100SW (84.78%). On the other hand, the poorly heritable traits are leaf length (5.92%), LCC (2.29%), NDF (6.93%) and NSP (1.68%). According to Ibrahim [17], a high heritability does not imply a high genetic advance for a particular quantitative trait, therefore, can be improved by selection.

The assessment of combining ability for water deficit showed that both non-additive and additive gene actions were key for tolerance to water deficit in cowpea. The high values of broad sense heritability for agronomic traits also demonstrated that additive and non-additive gene actions conferred resistance to water deficit conditions in cowpea. Additive variation plays an important role in the improvement of cowpea as a self-pollinated crop, and selection of best individuals in segregating population developed are possible via the presence of additive variation observed. Thus, the improvement of cowpea under the threat of climate change will help fight against hunger in the region and increase incomes of farmers and traders.

## Conclusion

Additive and non-additive genetic actions were observed in the traits studied. However, in both water regime conditions, additive gene action predominates over non-additive one for several traits including those related to grain yield. Therefore, these traits could be exploited by a pedigree selection method to obtain transgressive segregants. It was also observed in both water regime conditions that KVX61-1, IT06K242-3, IT07K-211–1-8, Kppodjiguèguè, IT99K-573–1-1, Tawa and IT97K-206–1-1 were good general combiners for proline content, chlorophyll content and traits associated with yield. This could be exploited as donor parents as they possess favorable alleles for drought tolerance and other traits directly associated with yield, thus could pass on high yield to their offspring. In addition, KVX61-1 × KVX396-18, IT06K242-3 × KVX396-18, IT07K-211–1-1 × KVX396-18, Kpodjiguèguè x KVX396-18, KVX61-1 × IT97K-206–1-1, IT06K242 -3 × IT97K-206–1-1, IT07K-211–1-1 × IT97K-206–1-1 and Kpodjiguèguè x IT97K-206–1-1 were found to be the best specific combiners for traits directly related to water deficit tolerance and yield. Number of days to pod maturity (NDPM), pod length (PL), number of pods per plant (NPP) and weight of hundred seeds (100SW) are found to be very highly heritable traits.

## Data Availability

The datasets used and analyzed during the current study are available from the corresponding author on reasonable request.
